# The Fly Pest

**Published:** 1921-05-21

**Authors:** 


*To the Editor of* THE HOSPITAL.

### The Fly Pest.

*To the Editor of* THE HOSPITAL.

SIR,—Anyone working in hospitals knows of this pest. Sir Alfred Yarrow, the most "practical man" I know, sent me lately a sprayer and some bottles of Harrison's "Fly Fright." You spray a window, or wherever flies are gathered together, and whether they are frightened or not I do not know, but they die at once. It can be got from Messrs. Harrison, Chemists, Haslemere. I have no interest in giving Messrs. Harrison this gratuitous advertisement, but I can, from experience, recommend this certain method of killing flies. I do not think it would harm linen. I intend to try it where flies are bothering patients.—Yours faithfully,

Kneesworth Hall,
Royston, Herts.

KNUTSFORD.

